# Asynchrony in Visual Consciousness and the Possible Involvement of Attention

**DOI:** 10.3389/fpsyg.2012.00314

**Published:** 2012-09-03

**Authors:** Konstantinos Moutoussis

**Affiliations:** ^1^Cognitive Science Division, Department of Philosophy and History of Science, University of AthensAthens, Greece

**Keywords:** attention, color, consciousness, functional specialization, motion, perception, perceptual asynchrony, vision

## Abstract

When subjects are asked to perceptually bind rapidly alternating color and motion stimuli, the pairings they report are different from the ones actually occurring in physical reality. A possible explanation for this misbinding is that the time necessary for perception is different for different visual attributes. Such an explanation is in logical harmony with the fact that the visual brain is characterized by different, functionally specialized systems, with different processing times for each; this type of organization naturally leads to different perceptual times for the corresponding attributes. In the present review, the experimental findings supporting perceptual asynchrony are presented, together with the original theoretical explanation behind the phenomenon and its implication for visual consciousness. Alternative theoretical views and additional experimental facts concerning perceptual misbinding are also reviewed, with a particular emphasis given to the role of attention. With few exceptions, most theories converge on the idea that the observed misbinding reflects a difference in perception times, which is in turn due to differences in neuronal processing times for different attributes within the brain. These processing time differences have been attributed to several different factors, attention included, with the possibility of co-existence between them.

## Functional Specialization and Perceptual Asynchrony in Vision

A picture of functional specialization with respect to the processing of different visual attributes has emerged from studies in the visual system (Zeki, 1978, 1993). Such a specialization makes sense since, for example, the perception of color involves very different computations from the ones involved in perceiving motion: motion perception requires the calculation of the way in which an object changes position in space over time, whereas the task of a system generating color would be to compare the energy-wavelength composition of the light reflected simultaneously from different objects in the field of view and thus calculate their reflectances, irrespective of any changes in the illumination (e.g., Land, 1971). Functional specialization states that these different tasks are undertaken by different, functionally specialized systems, occupying topographically separate locations in the visual brain (Zeki, 1978, 1993). Specialized brain areas are created in this way, each one characterized by specialized neurons with different connection patterns, different conduction velocities and so on. Such architecture of the visual brain begs the question of whether these separate systems could “finish” their so different tasks at exactly the same time. The word “finish” is used here to refer to the time necessary for the corresponding visual percept to emerge – I will refer to this as *perception time*. Given this distribution of function and the reasons for it, it seems likely that different visual attributes could have different perception times and are therefore not perceived in synchrony. It should be noted that we are currently unaware of the spatiotemporal structure of the neurobiological events underpinning visual awareness. Thus, perception time is regarded here as a property of the corresponding processing-perceptual system as a whole, free from any vague assumptions regarding particular visual areas and activation patterns in the brain. Since we are still far from understanding where or how a conscious visual percept arises in the brain, the exact relationship of the latter with activity reaching any certain levels in any particular brain areas (Moutoussis and Zeki, 2002; Moutoussis, 2009), back-projections and feedback (Lamme and Roelfsema, 2000), oscillations and synchronizations (Singer, 1999), etc., is beyond the interest of the general idea of perceptual asynchrony in vision.

The question of whether different visual attributes are perceived at exactly the same or at different times has been addressed experimentally (Moutoussis and Zeki, 1997a,b): If the perception time for a particular visual attribute, say color, is *dt* time shorter than that necessary for the perception time for another visual attribute, say motion, then the color which is present on the computer screen at time *t* will be perceived synchronously with the motion present on the screen at time *t−dt*. If this motion is different to the motion present on the screen at time *t*, then the color present on the screen at time *t* will not be perceived together with the motion with which it physically coexisted, but with a different one, which had occurred at time *t*−*dt*. Similarly, if the color changes at time *t* + *dt* then also the motion present on the screen at time *t* will be perceived together with this new color rather than with the color with which it occurred together in reality. By presenting stimuli changing both in color (red/green) and in motion direction (up/down) rapidly and continuously, and instructing participants to report which color-motion pairs were perceived as coexisting, color was found to be paired with the motion present on the computer screen ∼100 ms earlier (Moutoussis and Zeki, 1997a). The observed misbinding between the two visual attributes was taken to suggest that the color of an object is perceived roughly 100 ms before its direction of motion. We have called this phenomenon *perceptual asynchrony* and have put forward the idea that it is due to the different processing times necessary for the two functionally specialized systems to “finish” their corresponding tasks. Binding color and motion belonging to the *same* object is not crucial, since identical results were also obtained when color and motion were made to belong to *different* objects (Moutoussis and Zeki, 1997b)^1^. The perception of form (orientation) falls between that of color and of motion in time, with the estimated values of the three perception time differences adding up nicely (Moutoussis and Zeki, 1997b). The functionally specialized systems in vision thus seem able to create specific visual percepts in their own time and independently from each other, inspiring the so-called *microconsciousness theory*, which states that conscious visual perception is not single and unified but rather made out of several, independent consciousnesses of the different visual attributes (Zeki and Bartels, 1999).

The color-motion misbinding illusion has been verified by several studies (Arnold et al., 2001; Viviani and Aymoz, 2001; Arnold and Clifford, 2002; Nishida and Johnston, 2002; Bedell et al., 2003; Clifford et al., 2003, 2004; Moradi and Shimojo, 2004; Arnold, 2005; Holcombe and Cavanagh, 2008). Particularly interesting is a study that controls for response bias, by combining perceptual asynchrony and the color-contingent motion-after-effect (MAE; Arnold et al., 2001). Continuous presentation of a rotating stimulus for a period of time will make a subsequent static stimulus appear to rotate in the opposite direction (Mather et al., 2008). This after-effect can be made contingent on color, by associating a particular direction of motion to a particular color and the opposite direction to a second color, during the same adaptation period (Favreau et al., 1972). Thus, if during adaptation red is associated with rightward motion and green with leftward motion, a static red pattern will appear to rotate leftwards and a static green pattern to rotate rightwards. In this way, the characteristics of the after-effect will directly reflect the perceptual associations between color and motion during the adaptation period. Arnold et al. have used our psychophysical paradigm to adapt subjects to rotational motion, and then checked for a color-contingent MAE. If the perception of color and motion were veridical, the maximum MAE in their experiment would have been obtained when the color and motion oscillations are in phase, with no MAE when the two oscillations are 90° out of phase (both color-motion pairs equally present). However, results from this experiment were in accord with the existence of a perceptual lag between motion and color perception, as originally reported by Moutoussis and Zeki (1997a,b). Since there was no binding-task involved, this novel setup has the advantage of being protected against any possible response bias of the participants^2^. In an attempt to account for the observed MAE without accepting the existence of perceptual asynchrony, Johnston and Nishida (2001) have suggested a hypothetical and somewhat far fetched mechanism, by which a change in the firing rate of neurons during the initial and the final stage of the color stimulus results to asymmetrical adaptation. Even if such a hypothetical mechanism exists, it is still not so clear why the binding should be stronger during the first part of the appearance of the new color rather than that of the new motion.

## Alternative Views on the Misbinding Effect

As noted above, since we do not yet know where, when and how in the brain a visual percept is created, the time taken from the presentation of a stimulus to its conscious perception is unknown. The psychophysical paradigm of Moutoussis and Zeki (1997a,b) can potentially measure *perception time differences* between different visual attributes, since *perception times* *per se* cannot be directly measured. What can be directly measured, however, is the *reaction time* to a visual stimulus. Different studies have used different methods to compare reaction times to color and motion stimuli, giving varying results: one study reports a quicker response to color than to motion (Barbur et al., 1998), whereas no difference was found in another (Nishida and Johnston, 2002). On top of that, there is the problem of whether one should expect reaction time data to reflect any underlying differences in perception time. It is not necessarily correct to use reaction time data to isolate the perceptual component of the delay, since these data are confounded with both the preparation and the execution of a motor response. For example, one could equate the second part of a theoretical *stimulation-perception-decision-reaction* model between color and motion reaction time data, in order to draw inferences regarding the first part. But it is far from clear whether different, functionally specialized, systems share common decision mechanisms or access the motor system in the same way and thus equating the *perception-decision-reaction* part is questionable. It is also possible that *stimulation-reaction* shortcuts might sometimes bypass the stage of conscious perception for a quicker response to, say, stimuli which are in motion. Thus, although the time necessary for conscious perception is usually part of the reaction time to a stimulus, a straightforward inference from the latter to the former is not always possible (see Arnold, 2010 for similar arguments). The suggestion that motor responses could be based on the first incoming spikes, whereas perception integrates over a longer time period (Eagleman, 2010) could be a possible explanation for the discrepancy observed between the timing of perception and action. Nevertheless, and despite the fact that negative results (i.e., *not* finding a difference) are of secondary importance in general, the failure of Nishida and Johnston (2002) to find any differences in the response times to *specific* colors and motion directions, remains an open question for the perceptual asynchrony theory.

In addition to reaction time studies, the results on temporal order judgments (TOJ) between color and motion changes also vary^3^: some have found TOJs to be accurate (Nishida and Johnston, 2002; Bedell et al., 2003), whereas others have reported that color changes seem to precede synchronous motion onsets (Viviani and Aymoz, 2001; Aymoz and Viviani, 2004) or direction reversals (Adams and Mamassian, 2004). In general, the exact task performed by the subjects seems to be crucial: it has been shown that, using the exact same stimuli, TOJ tasks can yield no asynchrony when perceptual pairing judgments tasks do (Bedell et al., 2003; Clifford et al., 2003). Another issue is that of the rate of alternation: when participants were asked to judge which feature (color or motion) changed first, and the peak relative timing for synchronous judgment (TOJ choices equally split at 50% for each attribute) was taken as an indication of the perception time difference, the observed color-motion misbinding was diminished at slow alternations rates (Nishida and Johnston, 2002). But this result is perhaps not so surprising, since the nature of the task in the original experiment requires a moderately high alternation rate (1–2 Hz) for perceptual asynchrony to be revealed: only then does the perception time difference shift the temporal relation of the two percepts a significant proportion of the oscillation period, leading to a noticeable change in pairing. The phenomenon is thus diluted for very slow oscillations, but for moderate rates perception time difference is found to be independent from the rate of the oscillations (Moutoussis and Zeki, 1997a; Bedell et al., 2003; Holcombe and Cavanagh, 2008). If, on the other hand, the frequency of the oscillations is too high, perceptual pairing is an impossible task. This is the reason why, in a study using rapid alternation rates (between 3.6 and 5.3 Hz), delaying color changes was found to have no effect in color-motion pairing facilitation (Moradi and Shimojo, 2004). But if the rates used are within the range that makes the pairing task possible, delaying color with respect to motion facilitates perceptual pairing (Arnold, 2005).

In our original experiments (Moutoussis and Zeki, 1997a,b), both the color and the motion percept alternated between two values. Nevertheless, one could argue that, with respect to position, the motion change is “second-order” (a change in the way position changes over time – i.e., a change of a change) whereas the color change is “first-order” (just changing from one color to the other). It has been suggested that the observed misbinding is because the brain is slower in calculating a “second-order” change than it is in calculating a “first-order” change (Nishida and Johnston, 2002). Technically, two monitor-frames are necessary for a color change to take place, whereas a motion change needs three. This gives a ∼14 ms time advantage to color, which is far less than the ∼100 ms value observed experimentally. Furthermore, if memory is taken into account, the single next frame is enough to register a change for both color and motion. There is thus no big advantage for color in terms of the nature of the physical presentation, but perhaps brain mechanisms are internally biased (less sensitive?) against detecting a “second-order” change. Even so, it should be pointed out here that motion changes are “second-order” with respect to position, not to motion (we have a first-order change in directional motion). It is questionable whether motion *perception* can be reduced to nothing more than perceiving position changes over time. There are instances when motion can be perceived without perceiving any particular object changing position, as in random dot stimuli (Newsome et al., 1989), or even without any object changing position at all, as in the MAE (Mather et al., 2008) or the Leviant illusion (Zeki et al., 1993). Stimulating area V5 can induce the perception of motion, again without any particular object being observed to change position (Salzman et al., 1990). Such studies suggest that motion perception is an autonomous perceptual entity, rather than the first derivative of position with respect to time (for a review see Nakayama, 1985). In a series of experiments manipulating the stimuli so as to make the position change a first-order change (here/there) and the color to gradually vary from red to green in a sine-wave manner, the sign of the perceptual misbinding was reversed (Nishida and Johnston, 2002). Although such a result at first seems to support the *first* vs. *second-order* hypothesis, the nature of this experiment is quite different from the original one: instead of reporting the perceptual pairing between two color and two motion percepts, numerous colors and no motion percept at all were involved, and participants had to pair the *position* of an object to the *direction of change* of its color. In our experiments, on the other hand, as far as motion perception is concerned, there is a bimodal perceptual switch between two different percepts, exactly as is the case with color. It should also be noted that, in the Nishida and Johnston study, when comparing color and position changes of the same order (either first or second), positions were found to be paired with colors which were presented at a slightly later time (Nishida and Johnston, 2002). This, together with reports of incorrect pairing between first-order (color and orientation) changes (Moutoussis and Zeki, 1997b; Clifford et al., 2003), as well as between second-order changes (Arnold and Clifford, 2002 – see next section) dilutes significantly the strength of the *order-of-change* account for perceptual misbinding.

It could be that the *perception of the time* at which a percept was perceived (*when* was that?) could be different from the real time at which *perception of the percept* took place (*what* is that?) If so, a misbinding could emerge as a result of the meta-analysis of salient temporal features, by a neural mechanism dedicated to code the timing of events, suggesting that the subjective time course of visual experience is the product of analysis beyond the temporal processing of the content of the events themselves (Dennett and Kinsbourne, 1992; Nishida and Johnston, 2002). It is not so clear whether the idea of perceptual asynchrony is totally abandoned in such a theory, i.e., whether color and motion take the same time to be perceived or not. The theory rather concentrates on the hypothetical existence of an independent system in the brain, responsible for the perception of the time of events, which is different from the mechanisms responsible for the perception of the events themselves. In such a scenario, the psychophysically observed misbinding (Moutoussis and Zeki, 1997a,b) is no longer considered to be the result of perceptual mechanisms *per se*, but an inaccurate judgment of the time of occurrence of perceptual events. It reflects the properties of a third mechanism, which uses temporal markers to reference the time a specific event occurs in the world, rather than the time that the processing of the event is completed in the brain (Nishida and Johnston, 2002). An error-prone process of matching temporal markers of a different order (see above) could perhaps provide an alternative explanation to the perceptual misbinding observed. However, in addition to the ill-defined nature of temporal markers (see Arnold, 2010), one has to assume that, somewhere in the brain, a mechanism exists which is responsible and capable for timing perceptual events and providing the temporal order between them. Given functional specialization and no evidence for a terminal point of convergence in the brain (Shipp and Zeki, 1995), it would be a challenging task for this mechanism to have synchronous access to the output of several specialized processing-systems, in order to synchronize the perceived time of occurrence of percepts different in nature, and give an accurate picture of events in the real world.

Temporal markers are supposed to reference the time a specific event occurs in the world rather than the time the processing of this event completes in the brain. However, the idea of the perception of the time of a percept being different to the time that the actual percept is being perceived, seems quite awkward. It suggests a dissociation between the subjective time course of events, as it appears to the observer, and the times at which representations of those events are established in the observer’s brain. Even more awkward seems the idea of the brain being able to know about the timing of things happening elsewhere (i.e., in the outside world), something necessary for a mechanism to be able to correctly synchronize different perceptual events in order to reflect physical reality. It is already difficult enough to imagine how such a mechanism could know the exact timing of different events *within* the brain. Even if such a mechanism exists, its function seems more appropriate for TOJ tasks, reporting the temporal order of events. But TOJ and perceptual pairing are two very different tasks, not least because in the former participants need to make a decision *after* the presentation of the stimuli, based on the memory of single, transient perceptual events (see Viviani and Aymoz, 2001 or Gauch and Kerzel, 2008 for examples). On the contrary, in perceptual-binding, decisions are not based on memory, since the stimulus is continuously present on the screen and the subject has to decide *online* which color is being perceived together with which direction of motion. It has been suggested that “postdiction^4^” mechanisms could be involved in TOJs of single events, as for example in experiments investigating the flash-lag effect (Eagleman and Sejnowski, 2000). Therefore, using TOJ with respect to the instances at which color and motion changes occur, could potentially give misleading results with respect to the perception time of a particular visual attribute (see also the section on attention below). Finally, the independence of the apparent asynchronies on the oscillation rate (Moutoussis and Zeki, 1997a; Bedell et al., 2003) is problematic for a marker-mismatch account of the phenomenon (see Arnold, 2010 for a detailed argument), and it is far from clear why there should be a tendency to pair markers attached to first-order position transitions with markers attached to delayed (rather than earlier) color changes. In conclusion, the temporal marker theory remains, at least to me, highly speculative as well as problematic^5^.

Based on a possible “postdiction” character of visual perception in general, yet another alternative explanation of perceptual misbinding has been suggested (Moradi and Shimojo, 2004). The basic idea is that a postdictive analysis determines the perceptual properties of new surfaces, by waiting for ∼80 ms in order to integrate perceptual events taking place during this period and then allocating the result of this integration to its beginning. This time period is initiated by some sort of transient, like a direction reversal, which erases all previous information accumulated. The timing allocated by the brain to the result of perceptual integration is thus the commencement rather than the end of the integration period, something that could hypothetically compensate for the variability of neural transmissions (see Dennett and Kinsbourne, 1992). In this way, information from after an event is taken into account before committing to a visual interpretation (Eagleman and Sejnowski, 2000; Moradi and Shimojo, 2004). In an experiment using random dot stimuli with red and green dots, a particular group of dots suddenly turned gray and was set into motion, at the end of which these dots either returned to their original color or reversed color, and participants were found to report the color of the moving dots to be that *after* the motion was over (Moradi and Shimojo, 2004). This result was taken as an indication that the brain integrates perceptual events over a period of time, pairing together motion with a color that occurs at a later time, in a “postdiction” manner^6^. In the scenario in which a postdictive account of visual perception is combined together with the assumption that color is not treated evenly during the integration period (last part given more weight), perceptual asynchrony could perhaps find an alternative explanation (Moradi and Shimojo, 2004). However, an easy way to distinguish between this and the original brain-time explanation, is the fact that they predict different optimal conditions for making temporal judgments (see Arnold, 2005): postdiction gives a satisfactory explanation for the results observed at a phase difference of 90°, but cannot explain the results observed when the color and motion oscillations are in complete synchrony (i.e., at a phase difference of 0°). More specifically, if the appearance of a new direction of motion “resets” the system and makes the pairing between motion and color stronger during the later stage of this motion, then this could potentially explain why this motion is not equally paired with the two colors but more strongly with the second one at a phase difference of 90°. The same explanation would also predict, however, a perfect binding between motion and its corresponding color at a phase difference of 0°, something which is contrary to what has been observed (see text footnote 2). Perceptual asynchrony, on the other hand, not only explains equally well the result at a phase difference of 90°, but also predicts the misbinding observed at a phase difference of 0°. In a series of experiments in which the opposite direction of motion was replaced by a different transient (total absence of the moving stimulus), color-motion misbinding was minimized and motion–motion misbinding was induced (Arnold, 2005). As proposed previously (Arnold and Clifford, 2002), the absence of the opponent direction of motion seems to result in faster processing within the motion system, reducing its lag with respect to the color system and introducing a perceptual advantage compared to a situation in which the opponent direction is present. Postdiction, on the other hand, would predict that any change in the motion status resets the system, irrespective of the particular characteristics of this transient.

## The Effects of Processing Time Manipulations

If perceptual asynchrony is due to a difference between the processing times of different functionally specialized systems, changes in the speed of processing should lead to changes in the magnitude of asynchrony. Along this line of thought, the role of the well known physiological effect of motion opponency has been examined (Arnold and Clifford, 2002). In our original setup, the two motion directions used (up and down) activate neuronal populations which inhibit each other maximally (Barlow and Levick, 1965; Snowden et al., 1991), possibly leading to a significant delay in processing time within the motion system. Indeed, Arnold and Clifford (2002) have found that the magnitude of the perception time difference between color and motion varies with respect to the angular difference between the two directions of motion which are present in the stimulus. The maximum difference was observed when the two directions were opposite, i.e., when the inhibition between the two neuronal populations responsible for the processing of the motion signal was at its maximum. However, while reduced, a robust perceptual asynchrony was still evident in the presence of a relatively slight angular difference in motion direction, suggesting that direction-selective inhibition is not the sole cause of perceptual asynchrony. These results pose a problem for the Nishida and Johnston (2002) temporal marker account, since it is not clear why the position of a temporal marker signaling a given direction of motion should depend on the magnitude of the preceding direction change. Furthermore, the fact that it takes different amounts of time to perceive two different motion pairs, which are nevertheless both second-order changes (Arnold and Clifford, 2002), speaks against the *first-* vs. *second-order* explanation of asynchrony (Nishida and Johnston, 2002). This finding also suggests that visual experience does not require the mediation of interpretive processes (Dennett and Kinsbourne, 1992; Eagleman and Sejnowski, 2000) or the aim of any specialized temporal coding system (Nishida and Johnston, 2002). Similar results, showing a dependence of perception time differences on the relative directions of motion, have been also found using random dot stimuli (Bedell et al., 2003 – but see Amano et al., 2007 for objections and an alternative view on the directional-effect).

There is further evidence for a direct relationship between the time courses of sensory processing in the brain and the timing of perceptual events, coming from experiments that show a dependence of the magnitude of perceptual asynchrony on factors such as the salience of the stimuli (Adams and Mamassian, 2004), their luminance (Bedell et al., 2006), as well as their contrast and speed (Lankheet and van de Grind, 2010). Clifford et al. (2004) have manipulated depth, speed, and transparency to show that the phenomenology of binding parallels the physiological properties of area V5 (as is the case with direction-specific inhibition) and is thus a direct reflection of the time course of the underlying neural processing. It therefore seems that the magnitude of perceptual asynchrony varies in a manner that is broadly consistent with the known dynamics of sensory processing (see Arnold, 2010 for similar arguments). Within this neurobiological frame, a model explaining perceptual asynchrony with respect to feedback connections to V1 has been also proposed (Clifford, 2010), since the latter seems to be involved in both perceptual-binding (Hochstein and Ahissar, 2002; Shipp et al., 2009) and visual consciousness (Lamme and Roelfsema, 2000; Pascual-Leone and Walsh, 2001).

## The Possible Involvement of Attention

Attention is joined at the hip with visual perception and consciousness, the link being so strong that it is sometimes difficult to distinguish between them (see Lamme, 2004). Several lines of evidence suggest that attention could be involved in the integration of visual information (Treisman and Gelade, 1980; Reynolds and Desimone, 1999). More specifically, it has been suggested that attention plays a crucial role in feature pairing, by associating features at a particular spatial location (Treisman and Gelade, 1980) and constructing neurons with dual selectivity to color and motion, as revealed by both anatomical (Shipp et al., 2009) and neurophysiological (Croner and Albright, 1999) findings. Psychophysically, rapid alternations of color and motion (above 5 Hz) prevent their correct pairing, despite the fact that both are still individually identifiable (Moradi and Shimojo, 2004; Arnold, 2005). Such a low temporal resolution nicely fits with the idea that feature binding might be under the control of a slow, high-level process like attention (Duncan et al., 1994).

Given the above, it is possible that perceptual asynchrony could be influenced by attention, or even totally explained in terms of attentional mechanisms. If this is the case, manipulating attention should modulate the magnitude, and perhaps the sign, of perceptual asynchrony. Experiments show that, although attended changes appear to precede unattended ones in temporal judgments (Sternberg and Knoll, 1973; Reeves and Sperling, 1986), the effects of endogenous feature attention on perceptual asynchrony (as measured via errors in perceptual pairing) are not robust. In a study in which subjects were instructed to attend to a particular color and pair it with one of two possible orientations in half of the trials, while in the other half of the trials attend to a particular orientation and pair it with one of two possible colors (Clifford et al., 2003), the perception time advantage of color over orientation was decreased in 2/3 subjects when attending to orientation (compared to when attending to color). Such a result suggests that attention might be able to modulate the magnitude of perceptual asynchrony, perhaps by speeding up the processing of the attended attribute (Sternberg and Knoll, 1973; Posner et al., 1980; Stelmach and Herdman, 1991; Carrasco and McElree, 2001). Unfortunately, the small sample used in this study does not allow for any strong conclusions to be drawn. In a similar study, in which half the subjects were instructed to attend to color and the other half to motion, no difference in perceptual pairing was found between the two conditions (Arnold, 2005). However, using exactly the same methodology, an attentional effect has been reported in a meeting abstract some years ago (Enns and Oriet, 2004): asynchrony was found to reverse when subjects in one group were instructed to attend to color and then report the corresponding motion, compared to when subjects in a different group were instructed to attend to motion and report the corresponding color^7^. A weakness of this study is that only four phase differences between the color and the motion oscillations were used: 100% correlation (i.e., 0° and 180° phase differences) and 50% correlation (90° and 270° phase differences). The latter are quite difficult conditions (since color switches in the middle of the motion and vice versa) and a possible strategy to report the last segment of the non-attended stimulus could lead to the result reported. In a similar, recent study, in which many more phase differences were used and participants had to pair the color and the motion of peripherally presented random dot fields, attending to color vs. attending to motion did not alter perceptual misbinding in any significant way (Holcombe and Cavanagh, 2008). It therefore seems that, despite a few weak reports for the contrary, voluntary switching between feature dimensions cannot account for the better part of perceptual asynchrony.

Despite the fact that the effects of voluntary, endogenous attention are negligible, the possibility that involuntary, exogenous attention could play a role still remains. A straightforward way to modulate the ability of a stimulus to draw attention is to increase its saliency. Using a TOJ task, Adams and Mamassian (2004) showed that stimulus salience can indeed influence perceptual asynchrony magnitude, with more salient changes being perceived faster. In this study, saliency was measured in terms of performance in a previous change-detection task. Interestingly though, when the contrasted stimulus-changes were matched in terms of detection ease, color changes were still perceived as occurring before physically synchronous changes in direction. Thus, although exogenous attention seems able to modulate the magnitude of perceptual asynchrony, it cannot provide an adequate and complete explanation for it (Adams and Mamassian, 2004).

In a different study, strong external transients (known to be very effective in engaging attention – see Posner, 1980) were used, in order to modulate exogenous spatiotemporal attention (Holcombe and Cavanagh, 2008). It seems odd why spatial attention alone would give an advantage to any particular feature of the ones present in the particular spatial location, especially since it has been previously reported to be ineffective in changing perceptual asynchrony (Paul and Schyns, 2003). The temporal component seems to be more important here: perhaps a strong transient signal sent down both the color and motion pathways could somehow serve as a cue for synchronization, eliminating the asynchrony observed otherwise (Holcombe and Cavanagh, 2008). Generalizing this finding to the perceptual asynchrony reported by others (e.g., Moutoussis and Zeki, 1997a,b), Holcombe and Cavanagh argue that the latter could be due to unbalanced effects of intrinsic transients in the stimuli. Although they do not make absolutely clear what they mean by this, my personal understanding is that color changes could perhaps be more salient than motion ones, attracting attention first and thus leading to a more rapid processing of this attribute. Alternatively, they could simply mean that color changes are being processed more quickly than motion changes. Both of these explanation are not that far from the idea that, for one reason or the other, color is being processed faster than motion (Moutoussis and Zeki, 1997a,b). It is also not clear whether these authors discard functional specialization as the reason behind any differences in processing time of the two attributes, and their main point that attention operates on independent processing streams does not seem to be at odds with the functional specialization argument.

A distinction between the perception of the transients and the perception of the attributes themselves should be made here: in variations of our original experiment, in which subjects were asked to judge whether a color or a motion direction change occurred first, no perceptual asynchrony was observed (Nishida and Johnston, 2002; Bedell et al., 2003). The same is true for color and orientation changes: despite color showing a perception time advantage in the perceptual pairing task, no time difference was found between the perception of color changes and that of the orientation changes (Clifford et al., 2003). If anything, such findings suggest that both transients are made available to perception equally fast, and it is the actual calculation of what follows the change which takes longer in the case of motion. Also, if perceptual asynchrony results because of attention giving a start-advantage to color, do these authors implicitly suppose that the processing of color and motion take equal time? Given the difference between both the nature of these attributes and the properties, topographical distribution and organization of the brain mechanisms responsible for their processing, it is rather unlikely. A compromise would be to assume that there is a perception time difference due to different processing times, on top of which a modulatory role of (exogenous) attention is possible.

In addition to the theoretical issues discussed above, there are also some methodological ones in the Holcombe and Cavanagh (2008) study. In their experimental setup, random dot fields were arranged in a circular array around fixation. In each field the dots were oscillating between red and green, and between moving toward and away the fixation point, at various phase differences. While maintaining fixation, the attention of the subjects was automatically captured by the appearance of a luminance ring surrounding one of the fields. The task was to report the color and direction of motion of the dots inside this field, *during the presence of the ring*. What was found is that asynchrony was lost, and that report probability was independent for each feature and determined by how synchronized this feature was with the cueing ring. The authors concluded that the exogenous transient is much stronger than both the color and the motion transients and is thus the determining factor of what will be perceived and when. However, the cueing ring was presented very briefly (for half a period) and participants reported on the color and motion present during this short interval. Given the simultaneous presence of several stimuli, each one could be individually perceived only via (voluntary or involuntary) attentional selection. Stimuli were thus virtually presented only for a brief interval, during which a maximum of one change for each attribute took place – in most cases one of the colors or motion directions did not appear at all, leaving one of the attributes without a transient. In order to misbind something that is presented now to something that was presented earlier, you need something that was presented earlier! It is therefore not surprising that no misbinding is observed in cases where there are not enough stimuli present for misbinding to occur. It is possible that subjects perceived (not equally fast) the two attributes which were mostly available during this brief period, and reported them from memory (see below) when asked afterward, without any involvement of perceptual-binding whatsoever.

Another objection is that the task was *not* an *online perceptual pairing* between color and motion, but a *recall from memory* of the *presence* of color and of motion during this very brief period^8^. Experiments reporting perceptual asynchrony give participants ample of time to observe the continuously alternating stimuli, and are asked to report on the perceptual co-existence of color and motion *at the time they are experiencing it*, since reporting *after* the completion of a perceptual event is vulnerable to post-perceptual biases. It is very difficult to perceptually pair attributes at single brief presentations, and there is only one such reported case (Linares and López-Moliner, 2006) but with presentations which are still quite longer than the ones used by Holcombe and Cavanagh. What can be accurately reported in such brief presentations is the order of the *single changes* in motion direction and color (Nishida and Johnston, 2002; Bedell et al., 2003), a task which is very different since TOJs on attribute-changes can be accurate even when continuous presentations lead to false perceptual pairings (Bedell et al., 2003; Clifford et al., 2003). Not surprisingly, when participants in the Holcombe and Cavanagh (2008) study were allowed to attend to the stimulus throughout the whole presentation, a perceptual asynchrony between color and motion was reported. In another variation of their experiment, asynchrony was also eliminated when the flash was continuously presented but stepping from one dot field to the other. However, the part of the oscillation “illuminated” each time by the flash was always the same, and was also presented at different spatial locations, making this setup perceptually equivalent to (several repetitions of) the single-flash condition^9^. These methodological issues, together with the fact that the main finding of Holcombe and Cavanagh (2008) is based on a *negative result*, from only 3 subjects that did not show much consistency between them (see Figure A1 in their manuscript), unfortunately weaken the conclusive strength of the potentially interesting effects reported.

## Philosophical Issues Regarding Perceptual Asynchrony

The theoretical context in which these psychophysical results are put is based on two, perhaps simplified, assumptions. Firstly, that there is a given (objective) time^10^ at which the processing of visual information leads to the creation of a conscious visual percept. Secondly, the time at which a subject is having a perceptual experience, is also the time that the experience is perceived to happen, i.e., each time I have a percept, I also perceive that it is happening *now*. Both these assumptions have been questioned (Dennett and Kinsbourne, 1992; Johnston and Nishida, 2001), but the alternative “solutions” offered are even more vague and unsatisfactory than the problems they are trying to solve^11^. Given these two assumptions, the fact that we perceive different visual attributes at different times, raises some interesting theoretical implications regarding visual consciousness and consciousness in general. Since the experiments were initially conceived as a consequence of functional specialization in the visual brain it was natural, given the results, to suppose that perceptual asynchrony reflects a difference in processing time between the different, functionally specialized, systems. Furthermore, the fact that these different visual attributes are perceived independently and in their own times, supports the possibility for these systems to be not only processing but also perceptual ones. Functional specialization is in this way extended to the world of phenomenology and qualia, giving rise to the idea of multiple visual consciousnesses coexisting in vision (see Zeki and Bartels, 1999 for a theoretical expansion of this idea). However, the introspective unity of consciousness begs the question of how do these visual percepts, which arise at topographically different parts of the brain, come together as a single experience. The problem goes beyond vision, as it also applies to the way in which different sensory modalities, as well as mental events in general, are combined into a single, unified consciousness. An obvious solution would be to assume the existence of some “executive” brain area to which all other areas report, a central stage at the end of a hierarchical chain of “importance.” Such a solution arises from the old intuitive assumption of a “spirit,” “ghost in the machine,” “single-self,” etc., existing above and supervising over the rest of the brain, spending its time by comfortably inspecting mental events projected for its delight on the stage of a “Cartesian Theater.” Against such an intuition, a series of interesting philosophical arguments fighting this essentially dualistic approach, as well as fighting against the illusion of the existence of a single “self,” have been made by Dennett (1991) and Dennett and Kinsbourne (1992). Additionally, the neuroanatomical reality does not support the presence of such a “brain within the brain,” where all the parallel distributed processing eventually comes together. There are, of course, examples of convergence and cross-talking in the brain, an example related to this discussion being the existence of cortical and subcortical regions that receive multisensory input. What is missing is an area where everything comes together – both function and thus information seem to be dispersed throughout the brain^12^. Thus there seems to be no terminal station in the brain, the architecture of which is characterized by a segregated organization principle, containing several functionally specialized modules that remain more or less separate (Zeki, 1974, 1978; Fodor, 1983). The perceptual asynchrony results support such a view and extend it to the specific domain of visual consciousness.

The question, however, remains: in order to perform the task, subjects need to combine together their color and motion percepts. Doesn’t this mean that the corresponding two pieces of neuronal information need to also *physically* come together at a common brain area? If not, does this imply that we are looking for a solution outside the neural substrate (see Johnston and Nishida, 2001)? One the one hand, the way in which localized activation contributes to and affects the prevailing brain state and thus consciousness, remains unknown. On the other hand, the prevailing brain state is nothing more than the collection of these activations, what is happening *now* at various parts of my brain. We have previously proposes a bold, perhaps extreme solution (Moutoussis and Zeki, 2004), suggesting that there is no binding at all in consciousness but rather that different experiences are phenomenally “bound” together in virtue of an external factor, namely the time at which they occur. In this way, brain-time is still important for the when of a percept, without the necessity of a single brain structure that is critical is critical for perceptual-binding. It is perhaps an illusion that I am the same person perceiving both color and motion, and it is perhaps even more wrong to make a distinction between the “person” and the “percept.” If we instead assume that a “person” is nothing but a temporary composition of different mental events coexisting at a given point in time, would the problem be solved? One would still have to explain the way mental events are grouped and experienced together – why isn’t the color I perceive now bound to the motion that you perceive now? It probably has something to do with the fact that some groups of mental events (*my* mental events) are sharing a common brain (*my* brain), but as long as the relationship between the latter and the so-called “mind” remains a mystery, questions like this will also remain unresolved. However, it is important to point out that these problems do not arise because of assuming the presence of functional segregation in visual consciousness. Alternative views, suggesting that everything is done everywhere (Schiller, 1997), or that special areas supervise mental events and assign temporal markers to them (Nishida and Johnston, 2002), are equally subject to the problem of implementation with respect to this marvelous physical entity living inside our head.

## Conclusion

There seems to be good evidence for a relationship between the time courses of sensory processing in the brain and the perceived timing of perceptual events. Most accounts of the perceptual misbinding between color and motion accept this idea, the difference between them being the question of what it is that causes these processing time differences to occur. Even the temporal marker-matching theory, which began as a totally different approach, accepts in its latest, modified version (Amano et al., 2007) the existence of processing time differences at the heart of the phenomenon, and thus transforms the temporal marker account of perceptual asynchrony into yet another form of “brain-time” (see Arnold, 2010). With respect to attention in specific, no strong conclusion regarding its significance in the misbinding observed between different visual attributes can be drawn. Most studies have found weak (if any) effects, and the ones showing an effect are confounded by methodological issues. Furthermore, the finding that implicit processing manifests a similar asynchrony to conscious report (Arnold et al., 2001), argues against an explanation of perceptual asynchrony based entirely on attentional mechanisms. Thus, attention does not seem to be responsible for the best part of perceptual asynchrony in vision. The idea that the latter emerges as a direct consequence of functional specialization in the visual system, comes out as the most attractive explanation of the asynchrony phenomenon. Attention might be able to slightly alter the magnitude of the effect in favor of one or another attribute, and the differentiation between perceptual asynchrony being caused by differences in processing time vs. attention seems arbitrary, as the latter could very well influence the former. A model in which several factors (attention included) could influence the time necessary for neuronal processing, seems to be the most appropriate explanation for the perceptual asynchrony observed. However, the question of how different visual attributes, which are processed independently, are perceptually bound together to form a coherent conscious percept, remains open.

## Conflict of Interest Statement

The author declares that the research was conducted in the absence of any commercial or financial relationships that could be construed as a potential conflict of interest.

## Footnotes

^1^It should be noted here that perceptual asynchrony occurs only when continuous changes are apparent as happening within clearly separate objects: perceptual misbinding was not produced by manipulations making the stimuli appear as (continuously present) transparent objects (Clifford et al., 2004; Moradi and Shimojo, 2004)

^2^In their original study, Moutoussis and Zeki (1997a) have used confidence ratings to protect their findings against any possible response bias explanation: participants were found to be most confident of their pairing response when color and motion were physically out of phase.

^3^Rather than having to perceptually pair the perception of a particular color to that of a particular direction of motion, subjects in a TOJ task have to report whether a (usually) single change in color preceded a (usually) single change in motion or vice versa.

^4^This term is given to the idea that perception, rather than being online, accumulates evidence over small time intervals before committing to a given perceptual experience.

^5^For a recent defense of this theory, see Nishida and Johnston (2010)

^6^Note that these results can be equally well explained by perceptual asynchrony, a quicker perception time making color to be perceived together with motion that took place earlier in real time.

^7^It should be noted here that this abstract was never followed up by a proper journal article. In a recent publication by Enns et al. (2010), it is actually acknowledged that perceptual asynchrony is not simply the result of a “sluggishness” in attentional shifts from one attribute to the other.

^8^Note that, since there was enough presentation time for a single color-motion “pair” only, participants chose between four possible response buttons (rather than between two, as in most previous perceptual pairing experiments).

^9^The authors claim that an apparent asynchrony was also observed when color and motion sequences were presented only during a ring flash period (but without the ring). This would be an important finding but, unfortunately, no data is presented to support it.

^10^This time is probably not dimensionless – it should be considered as a range rather than as a time-point.

^11^The basic idea is that perhaps ‘the representation of sequence in the stream of consciousness is the product of the brain’s interpretive processes, not a direct reflection of the sequence of events making up these processes’ (Dennett and Kinsbourne, 1992).

^12^Even if such an area existed, an interesting philosophical game is the idea that, if different parts of it carried different bits of information, there would have to be yet another round of convergence, everything coming together to an even smaller part of this area and so on. When is one satisfied that all has come together? Is a new grandmother-cell type solution, where all information available in the brain would converge to a single “hyperneuron,” what we really want here? Therefore, it seems that a convergence-type solution to the binding problem creates nothing more than vicious cycles, and should perhaps be abandoned as a theoretical approach altogether.
